# Effects of metformin on human gingival fibroblasts: an in vitro study

**DOI:** 10.1186/s12903-023-02978-0

**Published:** 2023-05-15

**Authors:** Nouf Alshibani, Reem AlKattan, Eman Allam, Fahad A. Alshehri, Manal Matouq Shalabi, Nada Almuhanna, Houriah Almarshad, Anfal Aljamili

**Affiliations:** 1 Department of Periodontics and Community Dentistry, College of Dentistry, King Saud University, Riyadh, Saudi Arabia; 2 Oral and Dental Research Division, National Research Centre, Cairo, Egypt; 3 Department of Periodontics, College of Dentistry, Princess Nourah Bint Abdulrahman University, Riyadh, Saudi Arabia; 4 Saudi Board of Periodontics, Ministry of Health, Riyadh, Saudi Arabia; 5 Saudi Board of Endodontics, Ministry of Health, Riyadh, Saudi Arabia; 6 Prince Sultan Military Hospital, Madinah, Saudi Arabia

**Keywords:** Matrix metalloproteinases, Metformin, Human gingival fibroblasts, Periodontal diseases

## Abstract

**Objective:**

To investigate the effects of metformin (MF) treatment on the matrix metalloproteinases (MMPs) and proinflammatory cytokines production from lipopolysaccharide (LPS) - stimulated human gingival fibroblasts (HGFs).

**Methods:**

HGFs were obtained from subcultures of biopsies from clinically healthy gingival tissues of patients undergoing oral surgeries. Cell cytotoxicity assay was used to determine the effect of different concentrations of MF on viability of HGFs. HGFs were then incubated and treated with different concentrations of MF and Porphyromonas gingivais (Pg) LPS. MMP-1, MMP-2, MMP-8, MMP-9, IL-1β, and IL-8 expression analysis was performed using xMAP technology (Luminex 200, Luminex, Austin, TX, USA). Student’s t-test for a single sample was used to compare the mean values of the study groups with the control value. A p-value of <0.05 and 95% confidence intervals were used to report the statistical significance and precision of mean values.

**Results:**

Concentrations of 0.5, 1- and 2-mM MF had a minimal non-significant cytotoxic effect on the HGFs and caused statistically significant reduction of MMP-1, MMP-2, MMP-8 and IL-8 expressed by the LPS-stimulated HGFs.

**Conclusion:**

The results of the present study confirm that MF suppresses MMP-1, MMP-2, MMP-8 and IL-8 in LPS-stimulated HGFs suggesting an anti-inflammatory effect of MF and potential adjunct therapeutic role in the treatment of periodontal diseases.

## Introduction

Periodontal diseases have been considered the second most common oral disease affecting mankind since ancient times. Reports indicate that about 40–90% of the global population is affected by periodontitis, making it a significant worldwide public health problem. Periodontal diseases are the results of infections and inflammation of the gingival and bone tissues that surround and support the teeth. The inflammatory process is triggered by the plaque biofilm that accumulates on teeth surfaces. It is now well established that the pathogenesis of the disease is multifactorial and that the resultant tissue and bone loss occurs as a consequence of various interactions between the host immune response and the pathogenic bacteria [1–3]. The main infectious agents involved in the pathogenesis of periodontal diseases are mainly gram-negative anaerobic bacteria such as Porphyromonas gingivalis (Pg), Tannerella forsythia, and Aggregatibacter actinomycetemcomitans [4, 5]. Subsistence of these bacteria elicits the immune response characteristics of periodontal diseases associated with the release of several cytokines and enzymes that are largely responsible for the destruction of the gingival tissues and alveolar bone. One of the key host factors involved in these complex host immuno-inflammatory reactions is matrix metalloproteinases (MMPs) [6, 7].

MMPs are a family of structurally related but genetically distinct proteolytic enzymes that mediate the degradation of extracellular matrix (ECM) macromolecules, including interstitial and basement membrane components and are thus involved in various physiological and pathological conditions. Several MMPs have been recognized as main inflammatory and immune response regulators in periodontal diseases. Besides being involved in the regulation of the destructive periodontal inflammatory response, they are broadly responsible on the degradation of ECM and basement membrane components [7, 8].

The oral microbiome is extremely complex with the average adult harboring about 50–100 billion bacteria in the oral cavity representing about 200 predominant bacterial species. There is a strong correlation between the composition and diversity of oral microbiota and periodontal diseases. In addition, the interaction between the oral microbiota and the host immune response is a critical determining factor in the development and progression of periodontal diseases. The advancements in DNA sequencing and other molecular techniques have allowed researchers to better understand the compositional changes that occur in subgingival biofilms in the transition from health to gingivitis then to destructive periodontal disease. Full knowledge of the whole dynamic of the oral microbiota and its relationship with periodontal disease is crucial for improving diagnostics and setting new effective treatment strategies, such as targeted antimicrobial therapy and probiotics [9, 10].

Human gingival fibroblasts (HGF) are the predominant cell type in the periodontal connective tissue. These cells produce the components of the ECM, as well as the enzymes that degrade the ECM. The degradation of the collagen-containing tissues at the dento-epithelial junction initiates the formation of a periodontal pocket. This can eventually result in an inflammatory periodontal disease, which is characterized by a significant reduction in collagen fiber density in the gingival tissues, loss of Sharpey’s fibers and apical migration of the junctional epithelium. HGFs play a significant role in the pathogenesis of periodontal diseases. Regulation of HGFs cellular reaction is considered a crucial pathway in controlling the progression of periodontal tissues pathological alterations as well as healing processes [11, 12].

Metformin (MF) is the most commonly used oral anti-diabetic drug used to treat type 2 diabetes. It is considered a safe and well-tolerated medication that was first approved for clinical use in 1985 in the United Kingdom. MF acts by lowering plasma glucose level, inhibiting glucose production from the liver and reduction of glucose absorption by the intestinal cells. Besides its anti-diabetic hypoglycaemic activity, studies reported that it has other beneficial effects such as antimicrobial, osteogenic, antiendometriotic and antiatherogenic effects [13–18].

The purpose of this study was to investigate the effect of MF on the MMPs, IL-1β and IL-8 production from HGFs to gain insights into the mechanisms by which MF may alter the gingival tissue response and explore its potential anti-inflammatory role in periodontal diseases. Effects of MF were tested in vitro on cultured HGFs, in the presence or absence of Pg lipopolysaccharide (LPS).

## Methods

Subjects who participated in the study were provided with a brief explanation of the details of the research and signed informed consent for participation. The study was conducted in accordance with the principles embodied by the 1975 Declaration of Helsinki and approved by the Institutional Review Board (IRB) of King Saud University (project number: E-18-3318).

### Cell Collection and Culture

HGFs were obtained from clinically healthy gingival tissues from 10 different patients undergoing oral surgeries at College of Dentistry, King Saud University Hospital, Saudi Arabia. A 2 mm collar of gingival tissue was taken from the premolar or molar tooth indicated for crown lengthening. The tissues appeared healthy on clinical examination with no bleeding on probing and were further examined histologically. All procedures were done by the same operator (NA).

Tissues were then transported from the clinic to the laboratory in phosphate-buffered saline (PBS) solution, washed with 70% ethanol and rinsed in PBS several times to remove the ethanol then the tissues were minced into small fragments of approximately 1 mm^3^. The tissue pieces were placed in cell culture dishes, air dried, and incubated for 5–7 days at 37 °C and 5% CO_2_ in low-glucose (100 mg/L) Dulbecco’s modified Eagles medium (DMEM) supplemented with 15% fetal bovine serum, 200 mM L-glutamine, 100 U/mL penicillin and 50 µg/mL gentamycin. The cells that grew out of the explants were subcultured and maintained. Cells at passages four to eight were used in the experiments [19, 20].

### Cellular cytotoxicity assay

Cell cytotoxicity was assessed by using MTT (3- [4,5-dimethylthiazol-2-yl]-2,5-diphenyl tetrazolium bromide)-based cytotoxicity assay. HGFs cells were seeded in 96-well plates at density 2 × 10^5^ cell/well in 100 µl optimized medium. The total number of cells was determined by the trypan blue exclusion test (0.4%) using a cell counter. Cells were allowed to settle for 24 h before treatment with individual serial concentrations of MF. Except for the control, cells were treated with 0.5, 1 and 2mM concentrations of MF (Sigma-Aldrich, Steinheim, Germany) and allowed to grow further for 48 h. At the end of the incubation period and concentration point, 20 µl of Cell Titer 96® Aqueous One Solution Cell Proliferation Assay (Promega, Madison, WI, USA) was added at 37°C at the final concentration of 5 mg/ml. The 96-wells plate was kept in dark for 2 h. The optical density (OD) of each treatment was measured at an absorbance value of 490 nm using a microplate reader and was performed in four replicates. Values of ODs were normalized according to the control (untreated cells). Therefore, cell viability values of untreated cells were 100% while values of treated cells had values below 100% where the mean background values were subtracted from each value. The MF concentration lethal to 50% of cells was calculated from the dose-response curve.

### Cells treatment with Metformin

HGFs (150,000 cells per ml using Haemacytometer) were seeded in 3 ml plates and incubated in complete medium at 37 °C overnight. Except for the control sample, cells were treated with various concentrations of MF (0.5, 1, and 2 mM) for 24 h. Cells were also stimulated with 5 µg/m Pg LPS (InvivoGen, San Diego, CA) according to a previous study [19] for another 24 h (Table 1). Culture supernatants were then collected for the Milliplex analysis.

**Table 1:** Description of study samples and groups

Sample # (3 mL plate)	On Seeding	After 24 h
Sample 1	Control (0 MF)	-
Sample 2	MF (2 mM)	-
Sample 3	MF (1 mM)	-
Sample 4	MF (0.5 mM)	-
Sample 5	LPS (5 µg/ml)	-
Sample 6	MF (2 mM)	LPS (5 µg/ml)
Sample 7	MF (1 mM)	LPS (5 µg/ml)
Sample 8	LPS (5 µg/ml)	1 mM MF
Sample 9	LPS (5 µg/ml)	2 mM MF

### Milliplex analysis

MMPs and cytokines analysis was performed by using xMAP technology (Luminex 200, Luminex, Austin, TX, USA) to measure MMP-1, MMP-2, MMP-8, MMP-9, IL-1β, and IL-8. The Milliplex MAP multiplex assay Human MMP Panel 2 Magnetic Bead HMMP2MAG-55 K and HSP2MAG-63 K (Millipore, Billerica, MA, USA) kits were conducted in 96-well microplate format according to the manufacturer’s recommendations. Concentrations of MMPs were determined on the Bio-Plex Protein Array System (Bio-Rad, Hercules, CA, USA). Briefly, each of the bead solutions was transferred into a mixing vial and adjusted to a volume of 3 ml with bead diluents. Internal controls and standards were included in every assay. Following the addition of sample supernatants and beads, the resulting mixture was incubated overnight at 4 °C. The plate was read by Bio-Plex array reader with the Luminex 200 software system [20].

### Statistical analysis

Data were analyzed using SPSS 21.0 version (IBM Inc. Chicago, USA) statistical software. Student’s t-test for a single sample was used to compare the mean values of the study groups with the control value. A p-value of <0.05 and 95% confidence intervals were used to report the statistical significance and precision of mean values.

## Results

**Fig. 1:**
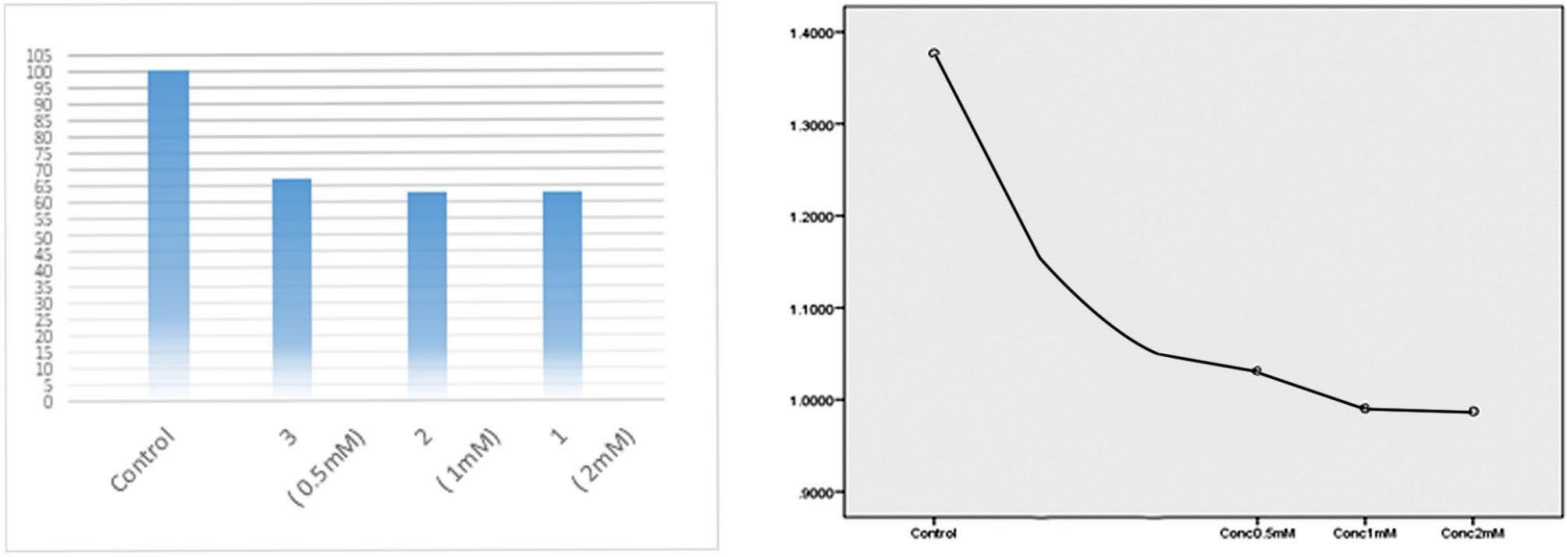
Effects of different MF concentrations on HGFs cell viability

Potential cytotoxicity of MF on cell viability was measured by MTT cell viability assay (Fig. 1). Different concentration groups of MF were tested (control, 0.5mM, 1mM, 2mM), each group was repeated three times. The mean of all groups was 1.09 ± 0.23 with 95% confidence interval (0.982–1.211). Measurement of potential cytotoxicity of MF on cellular viability showed a mean of MTT in the control group of 1.37, 1.03 at 0.5 mM of MF, 0.99 at 1mM of MF and 0.98 at 2mM of MF. No statistically significant difference existed between different MF concentrations on cellular viability (p = 0.31).

**Fig. 2:**
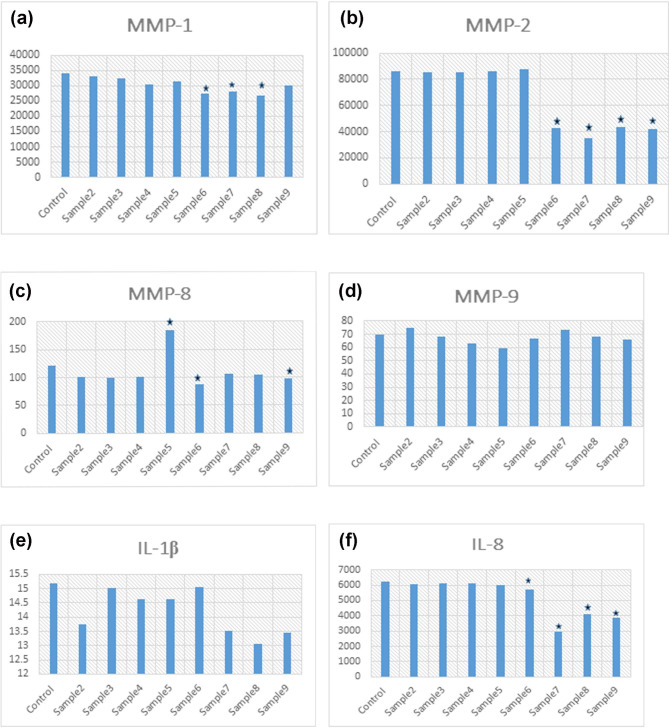
Mean and standard deviation of all groups for (**a**) MMP-1, (**b**) MMP-2, (**c**) MMP-8, (**d**) MMP-9, (**e**) IL-1β, and (**f**) IL-8.

The comparison of mean values of six outcome variables (MMP-1, MMP-2, MMP-8, MMP-9, IL-1β, and IL-8) in eight samples using MF treatment with control values shows statistically significant difference in the mean values of MMP1 for sample 6 (2mM MF + LPS), sample 7 (1mM MF + LPS) and sample 8 (LPS + 1mM MF), where the sample mean values were statistically significantly lower than the control value. The mean values of MMP-2 for sample 6 (2 mM MF + LPS), sample 7(1mM MF + LPS), sample 8 (LPS + 1mM MF) and sample 9 (LPS + 2mM MF) were statistically significantly lower than the control value. The mean value of MMP-8 for sample 5 (LPS) was significantly higher than the control value. The mean values of MMP-8 in sample 6 (2mM MF + LPS) and sample 9 (LPS + 2mM MF) were significantly lower than the control value. The mean values of IL-8 for sample 7 (LPS + 1mM MF) and 8 (LPS + 1mM MF) lower than the control sample with non-statistically significant values. For the outcome variables MMP-9 and IL-1β, none of the samples mean values were significantly different from the control sample (Table 2; Fig. 2).

**Table 2:** Comparison of mean values of MMP-1, MMP-2, MMP-8, MMP-9, IL-1β, IL-8 in all samples with the control value

Outcome variables	Mean values using Metformin	Control value	Difference in mean value	t-value	p-value
**MMP-1** Sample2 Sample3 Sample4 Sample5 Sample6 Sample7 Sample8 Sample9	32919.67 32310.67 30370.66 31531.00 27479.00 28111.33 26674.33 30044.33	34154.33 34154.33 34154.33 34154.33 34154.33 34154.33 34154.33 34154.33	-1234.66 -1843.66 -3783.66 -2623.33 -6675.33 -6042.99 -7479.99 -4109.99	-0.609 -1.364 -3.771 -0.982 -7.028 -5.012 -17.462 -2.579	0.604 0.306 0.063 0.429 0.019* 0.037* 0.003* 0.123
**MMP-2** Sample2 Sample3 Sample4 Sample5 Sample6 Sample7 Sample8 Sample9	85000.33 85175.66 86440.00 87454.66 42692.33 34762.00 43714.66 42102.00	85914.67 85914.67 85914.67 85914.67 85914.67 85914.67 85914.67 85914.67	-914.33 -739.00 525.33 1539.99 -43222.33 -51152.67 -42200.00 -43812.67	-0.494 -1.327 0.177 1.107 -158.551 -50.521 -54.857 -440.638	0.670 0.315 0.875 0.383 < 0.0001* < 0.0001* < 0.0001* < 0.0001*
**MMP-8** Sample2 Sample3 Sample4 Sample5 Sample6 Sample7 Sample8 Sample9	101.69 99.69 101.69 184.24 87.31 105.82 103.66 97.69	121.51 121.51 121.51 121.51 121.51 121.51 121.51 121.51	-19.813 -21.816 -19.813 62.730 -34.193 -15.689 -17.843 -23.820	-2.805 -4.019 -2.805 12.110 -16.256 -3.893 -2.4298 -11.619	0.107 0.056 0.107 0.006* 0.003* 0.060 0.135 0.007*
**MMP-9** Sample2 Sample3 Sample4 Sample5 Sample6 Sample7 Sample8 Sample9	74.90 67.80 62.99 59.60 66.39 73.20 68.09 66.05	69.79 69.79 69.79 69.79 69.79 69.79 69.79 69.79	5.11 -1.98 -6.80 -10.18 -3.40 3.41 -1.69 -3.73	1.730 -0.258 -2.000 -1.733 -1.000 0.999 -0.377 -1.150	0.225 0.820 0.183 0.225 0.422 0.422 0.742 0.369
**IL-1** **β** Sample2 Sample3 Sample4 Sample5 Sample6 Sample7 Sample8 Sample9	13.74 15.03 14.62 14.62 15.04 13.52 13.07 13.44	15.18 15.18 15.18 15.18 15.18 15.18 15.18 15.18	-1.43 -0.14 -0.56 -0.56 -0.13 -1.65 -2.10 -1.73	-1.836 -0.193 -1.515 -1.515 -0.250 -1.907 -1.812 -1.906	0.207 0.864 0.268 0.268 0.8257 0.196 0.211 0.196
**IL-8** Sample2 Sample3 Sample4 Sample5 Sample6 Sample7 Sample8 Sample9	6055.33 6094.66 6139.00 5990.66 5689.66 2952.00 4085.66 3885.00	6229.67 6229.67 6229.67 6229.67 6229.67 6229.67 6229.67 6229.67	-174.33 -135.00 -90.67 -239.00 -540.00 -3277.67 -2144.00 -2344.67	-1.460 -21.316 -0.900 -1.978 -7.914 -8.306 -11.37 -25.311	0.281 0.202 0.4627 0.186 0.015* 0.014* 0.008* 0.002*

*Statistically significant p <0.05

## Discussion

Periodontal diseases are characterized by prolonged inflammation and progressive destruction of the periodontal tissues as a result of the host immune response to the microbial plaque accumulation. The sustained release of the host proteolytic enzymes is primarily responsible for the associated collagen degradation and tissue damage. Diabetic patients are at increased risk of periodontal diseases since the hyperglycemia status significantly affects their immune, metabolic, and hemodynamic functions [21–23]. Studies showed that MF, the commonly used antidiabetic drug for improving insulin resistance, has a favorable effect on the periodontal tissue response to the pathogenic stimuli [24–26]. A randomized controlled clinical trial evaluated the efficacy of 0.5%, 1%, and 1.5% MF gel drug delivery system as an adjunct to scaling and root planning (SRP) for treatment of intrabony defects (IBDs) in patients with chronic periodontitis compared to a placebo gel. Their results showed that the local delivery of MF into the periodontal pocket caused significant reduction in probing depth, clinical attachment loss, and IBD depth compared to placebo in adjunct to SRP [24]. In a later study, the authors compared the effect of a 1% MF gel with placebo on IBDs in patients with chronic periodontitis without any systemic disease and concluded that the 1% MF significantly improved the clinical and radiographic parameters of the IBDs in those patients [25]. Another study investigated the effects of MF on the level of oxidative stress and bone loss reported that MF, at a concentration of 50 mg/kg, decreased the inflammatory response, oxidative stress and bone loss in ligature-induced periodontitis in rats [26].

A hallmark of periodontal attachment loss is the degradation of type I collagen as a result of MMPs release by the host’s resident cells in response to the inflammatory stimuli. The activity of MMPs is controlled by changes in the delicate balance between the expression and activation of the MMPs and their major endogenous inhibitors, the tissue inhibitors of matrix metalloproteinases (TIMPs). MMPs are expressed by a variety of periodontal ligament cells including mainly polymorphonuclear cells, fibroblasts, macrophages, and keratinocytes. MMPs are stimulated either directly by microbial products from the bacterial plaque that colonize the teeth and their surroundings such as LPS or indirectly by inflammatory mediators generated in response to oral microorganisms. LPS is the principal component of gram-negative bacteria that activates the innate immune system and Pg LPS is well known to be an important virulence factor in the mechanism of periodontal diseases [27–29].

In our study, HGFs were treated for 24 h with different concentrations of MF with and without LPS and showed heterogeneous results with regards to MMPs expression. The study also examined the effect of MF on IL-1 and IL-8 which are two main proinflammatory mediators that play important roles in periodontal diseases pathogenesis. Results indicated that MF alone had no significant effect on the expression of either MMPs or ILs. Esfahanian et al. reported that treatment with MF demonstrated a strong suppressive effect of mRNA levels of MMP-2 and − 9 in endothelial cells [30]. The difference in the described effect could possibly be explained by the fact that different types of cells react differently to the same stimuli.

On the other hand, the production of MMP-1, -2, -8 and IL-8 were significantly reduced when 2 mM of MF treatment was followed by 5% LPS treatment after 24 h. LPS alone increased MMP-8 expression significantly with a p-value of 0.006. We assume that the conditioning of HGFs with MF may have considerably masked the destructive effect of LPS specially on the expression of MMP-8. MMP-8 is a collagenase that has the unique ability to break down type I and III collagen which is critical for periodontal tissue destruction. A major persistent influx of neutrophils, the main cellular source of MMP-8, is characteristic of periodontal diseases. Interestingly, both MMP-9 and IL-1β release did not demonstrate any difference between MF treated various groups in comparison to the control group. These results confirm that MF, aside from its glycemic control effects, exerts a critical role in modulating the inflammatory response to periodontal pathogens.

Previous reports have similarly indicated that MF exhibited inhibitory effects on LPS-influenced inflammatory cytokines production in murine macrophages, human gingival and periodontal ligament cells [31–33]. Collectively, the results of this study indicate that MF modify the response of LPS-stimulated HGFs through suppression of inflammatory MMP-1, -2, -8 and IL-8. This may provide an insight on the mechanism by which MF could be beneficial controlling the inflammatory response in diabetic patients receiving the drug. The possible long-term in vivo effect needs to be further clarified by clinical studies.

## Conclusion

The results of the present in vitro study confirm that MF suppresses MMP-1, MMP-2, MMP-8 and IL-8 in LPS-stimulated HGFs suggesting a potential positive impact on gingival cells reducing inflammation and extracellular matrix degradation. Those anti-inflammatory effects of MF provide evidence for possible adjunct therapeutic role in the treatment of periodontal diseases. Future in vivo and clinical studies evaluating this link may aid in understanding the long-term efficacy of MF for periodontal diseases management and dose response impacts of MF.

## Data Availability

Data and materials are available upon request by mailing the first author, Nouf Alshibani (nalshibani@ksu.edu.sa)
